# Piezo2 expression and its alteration by mechanical forces in mouse mesangial cells and renin-producing cells

**DOI:** 10.1038/s41598-022-07987-7

**Published:** 2022-03-10

**Authors:** Yuki Mochida, Koji Ochiai, Takashi Nagase, Keiko Nonomura, Yoshihiro Akimoto, Hiroshi Fukuhara, Tatsuo Sakai, George Matsumura, Yoshihiro Yamaguchi, Miki Nagase

**Affiliations:** 1 Department of Anatomy, Kyorin University School of Medicine, 6-20-2 Shinkawa, Mitaka, Tokyo, 181-8611 Japan; 2 Department of Trauma and Critical Care Medicine, Kyorin University School of Medicine, Tokyo, Japan; 3 Kunitachi Aoyagien Tachikawa Geriatric Health Services Facility, Tokyo, Japan; 4 Division of Embryology, National Institute for Basic Biology, Okazaki, Japan; 5 Department of Urology, Kyorin University School of Medicine, Tokyo, Japan; 6 Juntendo University Faculty of Health Science, Tokyo, Japan; 7 Department of Anatomy and Life Structure, Juntendo University Graduate School of Medicine, Tokyo, Japan; 8 Department of Medical History, Juntendo University Faculty of Medicine, Tokyo, Japan

**Keywords:** Molecular medicine, Nephrology

## Abstract

The kidney plays a central role in body fluid homeostasis. Cells in the glomeruli and juxtaglomerular apparatus sense mechanical forces and modulate glomerular filtration and renin release. However, details of mechanosensory systems in these cells are unclear. Piezo2 is a recently identified mechanically activated ion channel found in various tissues, especially sensory neurons. Herein, we examined Piezo2 expression and regulation in mouse kidneys. RNAscope in situ hybridization revealed that *Piezo2* expression was highly localized in mesangial cells and juxtaglomerular renin-producing cells. Immunofluorescence assays detected GFP signals in mesangial cells and juxtaglomerular renin-producing cells of *Piezo2*^*GFP*^ reporter mice. *Piezo2* transcripts were observed in the *Foxd1*-positive stromal progenitor cells of the metanephric mesenchyme in the developing mouse kidney, which are precursors of mesangial cells and renin-producing cells. In a mouse model of dehydration, *Piezo2* expression was downregulated in mesangial cells and upregulated in juxtaglomerular renin-producing cells, along with the overproduction of renin and enlargement of the area of renin-producing cells. Furthermore, the expression of the renin coding gene *Ren1* was reduced by *Piezo2* knockdown in cultured juxtaglomerular As4.1 cells under static and stretched conditions. These data suggest pivotal roles for Piezo2 in the regulation of glomerular filtration and body fluid balance.

## Introduction

The morphology and function of tissues and organs, including intricate organs such as the kidney, are tightly coordinated, and mechanical forces are key factors that can mediate this coordination^1^. For instance, the functions of mesangial cells in the glomeruli and renin-producing cells in the juxtaglomerular apparatus are finely regulated by hemodynamic mechanical factors, which contributes to the homeostasis of the body^2^.

Glomerular endothelial cells, podocytes, and mesangial cells are three cellular components of renal glomerular tufts. The glomerular endothelial cells and podocytes control urinary filtration, whereas the functions of mesangial cells include (1) structural support, (2) secretion of extracellular matrix (ECM), (3) modulation of glomerular filtration through mesangial cell responses to vasoactive stimuli, (4) phagocytosis of macromolecules and immune complexes, and (5) response to and secretion of cytokines^3,4^. Among these, functions (1) and (3) suggest the importance of mechanical strain on the morphology and functions of mesangial cells. For example, mesangial cells and the glomerular basement membrane form a contracting framework, thereby supporting the integrity of the glomerular tuft and its capillary wall tension^5,6^. Mesangial cells also secrete ECM in response to mechanical stimuli, including collagens and fibronectin^7–10^. This point is clinically important since glomerulosclerosis is characterized by the uncontrolled proliferation of mesangial cells and an increase in ECM deposition^3^. However, the mechanism by which mechanical stimuli are initially transmitted to mesangial cells and the molecules that act as mechanosensors remain to be elucidated.

Renin secretion from the juxtaglomerular apparatus is a rate-determining factor of the renin angiotensin system, which controls blood pressure and body fluid balance^11,12^. Renin-producing cells in the juxtaglomerular apparatus (also known as juxtaglomerular cells, JG cells, or granule cells) are located in the medial layer of the afferent arterioles. Triggers of renin secretion from renin-producing cells include (1) baroreception by renin-producing cells that sense perfusing pressures of the afferent arterioles, (2) Cl^−^ concentration in the macula densa, and (3) neuroendocrine regulation, including β-adrenergic receptor activity. Although some candidate molecules, such as transient receptor potential vanilloid 4 (Trpv4) ion channel, have been considered^13^, the mechanosensing mechanisms of baroreception have not been fully investigated.

Morphologically, mesangial cells and renin-producing cells have the same embryonic origins^14,15^. The metanephros (renal primordia) comprises three progenitor groups. The ureteric buds differentiate into collecting ducts, the renal pelvis, and the ureteric epithelium^16^. *Six2*-positive cap mesenchyme differentiates into the entire nephron from podocytes to the distal tubules^17^. *Foxd1*-positive stromal cells differentiate into both glomerular mesangial cells and mural cells of the renal arterial tree (renin cells, smooth muscle cells, perivascular fibroblasts, and pericytes)^14,15,18^. However, it is not clear whether mesangial cells and renin-producing cells share a common mechanosensing system.

Piezo proteins, including Piezo1 and Piezo2, are now regarded as *bona fide* mechanically activated ion channels that mediate various biological phenomena^19^. Piezo1 is predominantly expressed in non-neural tissues, such as blood vessels^20,21^, where it regulates blood pressure and vascular development. Piezo2 is mainly expressed in neural sensory systems, such as the dorsal root ganglia^22^, sensory nerve terminals^22^, Merkel cells^23,24^, and muscle spindles^25^. Piezo2 mediates cutaneous light touch^22–24^, itching^26^, proprioception^25^, baroreception at the carotid sinus and aortic arch (together with Piezo1)^27^, and airway stretch^28^. In the urinary system, Piezo1 is expressed in the glomeruli, tubules, and collecting ducts^29,30^. Piezo2 also plays an important mechanosensing role in the bladder^31^. However, the distribution of *Piezo2* in the kidney, changes in *Piezo2* levels during development, and *Piezo2* expression regulation in response to altered mechanical load are still unknown.

In this study, we found that Piezo2 was expressed in glomerular mesangial cells and juxtaglomerular renin-producing cells at both the mRNA and protein levels. Embryonic expression of *Piezo2* was observed in *Foxd1*-positive stromal progenitor cells and their derivatives but not in *Six2*-positive nephron progenitors. In response to the reduction of mechanical load to the glomeruli by dehydration, *Piezo2* expression was downregulated in mesangial cells, whereas its expression was upregulated in juxtaglomerular renin-producing cells, along with an overproduction of renin. *Piezo2* knockdown in cultured mouse As4.1 cells, juxtaglomerular cell line, showed significant inhibition of the renin coding *Ren1* gene expression under static and stretched status. These data suggest important roles of Piezo2 in the physiological regulation of body fluid homeostasis.

## Results

### *Piezo2* transcripts are localized in mesangial cells and juxtaglomerular renin-producing cells

**Figure 1:**
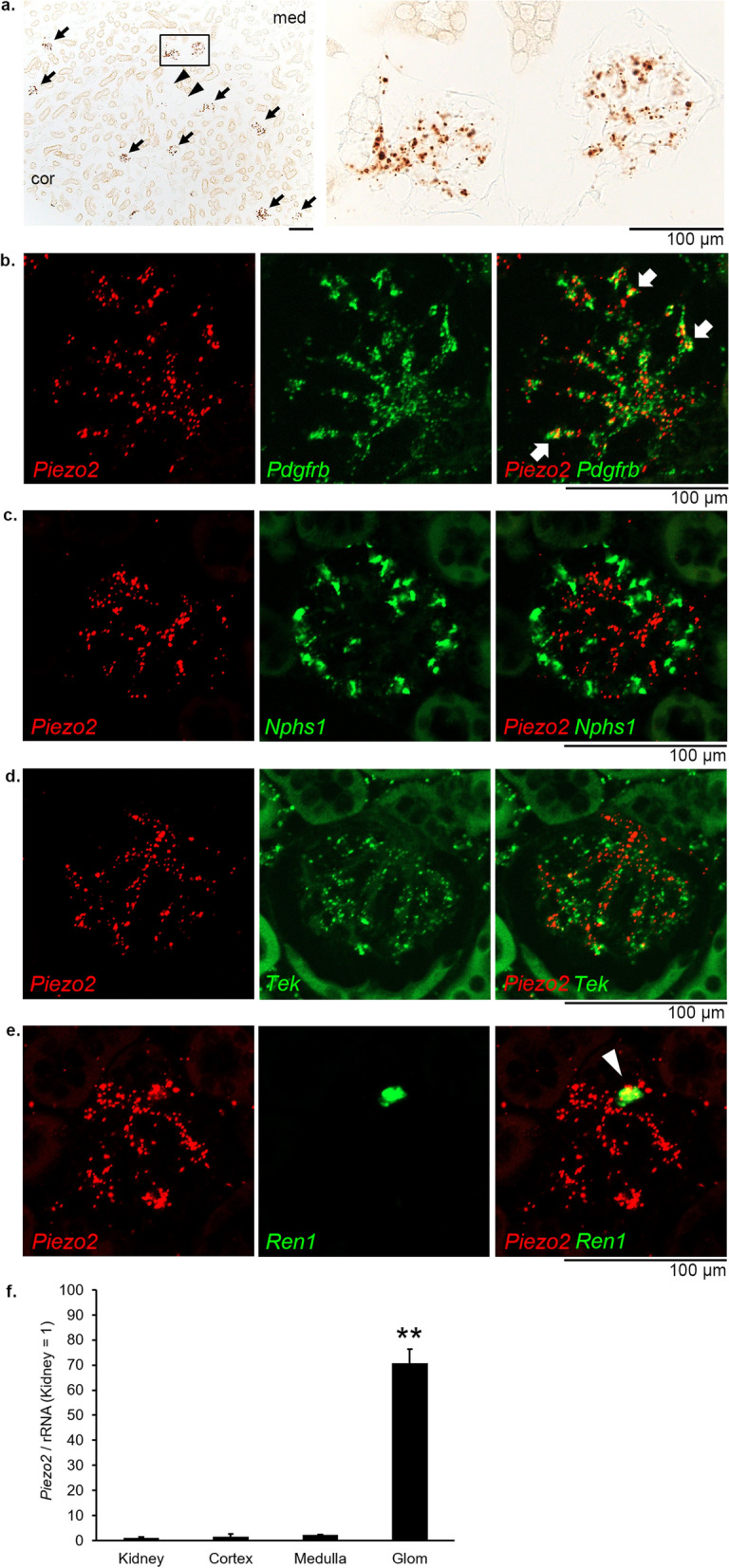
Gene expression and localization of *Piezo2* in the kidneys of adult C57BL/6J mice. (**a**) RNAscope in situ hybridization of *Piezo2*. Signals were visualized as brown dots by DAB chromogen detection. *Piezo2* is expressed specifically in the glomerular and juxtaglomerular regions (arrows). The right panel shows a magnified view of the boxed area in the left panel. Arrowheads indicate vessels. cor, cortex; med, medulla. Scale bars, 100 µm. (**b**–**e**) Fluorescence double RNAscope in situ hybridization of *Piezo2* (red) and cell markers (green), *Pdgfrb* (a mesangial cell marker), *Nphs1* (a podocyte marker), *Tek* (an endothelial cell marker), and *Ren1* (a marker for renin-producing cells). Arrows indicate *Piezo2*-expressing *Pdgfrb*-positive mesangial cells. Arrowhead indicates *Piezo2*-expressing *Ren1*-positive renin-producing cells. Scale bars, 100 µm. (**f**) Quantitative RT-PCR analysis of *Piezo2* in the kidney, cortex, medulla, and glomerular (Glom) fractions. *Piezo2* expression in the kidney is arbitrarily expressed as 1. Bars represent mean ± s.e.m. **P < 0.01 vs. kidney, one-way ANOVA followed by Bonferroni's post hoc test (*n* = 4 per group).

To examine the expression patterns of *Piezo2* transcripts, we first performed in situ hybridization using RNAscope technology. Even a single mRNA molecule can be visualized as a dot using RNAscope^32^. In the normal C57BL/6J mouse kidney, DAB-stained brown dot signals representing *Piezo2* transcripts were specifically observed in the glomeruli (Fig. 1a, arrows). No apparent dot signals were detected in tubular or duct cells. There were no specific dot signals when the negative control probe targeting the invertebrate gene dihydrodipicolinate reductase (*dapB*) was used (Supplementary Fig. S1a). The brownish staining, not dot signals, observed in some tubules was considered as background staining. Ubiquitous dot signals were observed for the positive control mouse housekeeping gene peptidylprolyl isomerase B (*Ppib*, Supplementary Fig. S1b). Additionally, dot signals for *Piezo2* mRNA were detected in the nodose ganglion in a pattern similar to that reported by Zeng et al.^27^, serving as another positive control (Supplementary Fig. S1c–e). These data validate the applicability of the RNAscope technology in our study.

To characterize the *Piezo2*-positive cells more precisely, we next performed double RNAscope in situ hybridization of *Piezo2* with cell marker probes using fluorescent dyes. Within the glomerular tufts, *Piezo2*-positive cells were mostly co-expressed with the platelet-derived growth factor receptor, beta polypeptide (*Pdgfrb*) gene, a marker for mesangial cells^33,34^ (Fig. 1b). On the contrary, co-expression of *Piezo2* transcripts with the podocyte marker *Nphs1* (also known as nephrin) was not observed (Fig. 1c). *Piezo2* transcripts were only rarely colocalized with transcripts for the endothelial marker *Tek* (Fig. 1d). Notably, *Piezo2* transcripts were detected in *Ren1*-positive cells in the juxtaglomerular apparatus (Fig. 1e), indicating *Piezo2* expression in the renin-producing cells within the afferent arteriolar walls.

We further evaluated the distribution of *Piezo2* mRNA using real-time quantitative RT-PCR of kidney tissues and fractions. Consistent with the staining pattern, *Piezo2* expression was concentrated in the glomerular fraction, compared with the low expression in the whole kidney, cortex, and medulla fractions (Fig. 1f). Taken together, these data indicate that *Piezo2* mRNA in the kidney is primarily expressed in mesangial cells and renin-producing cells.

### Localization of Piezo2-GFP fusion protein indicates Piezo2 expression in mesangial cells and renin-producing cells

**Figure 2:**
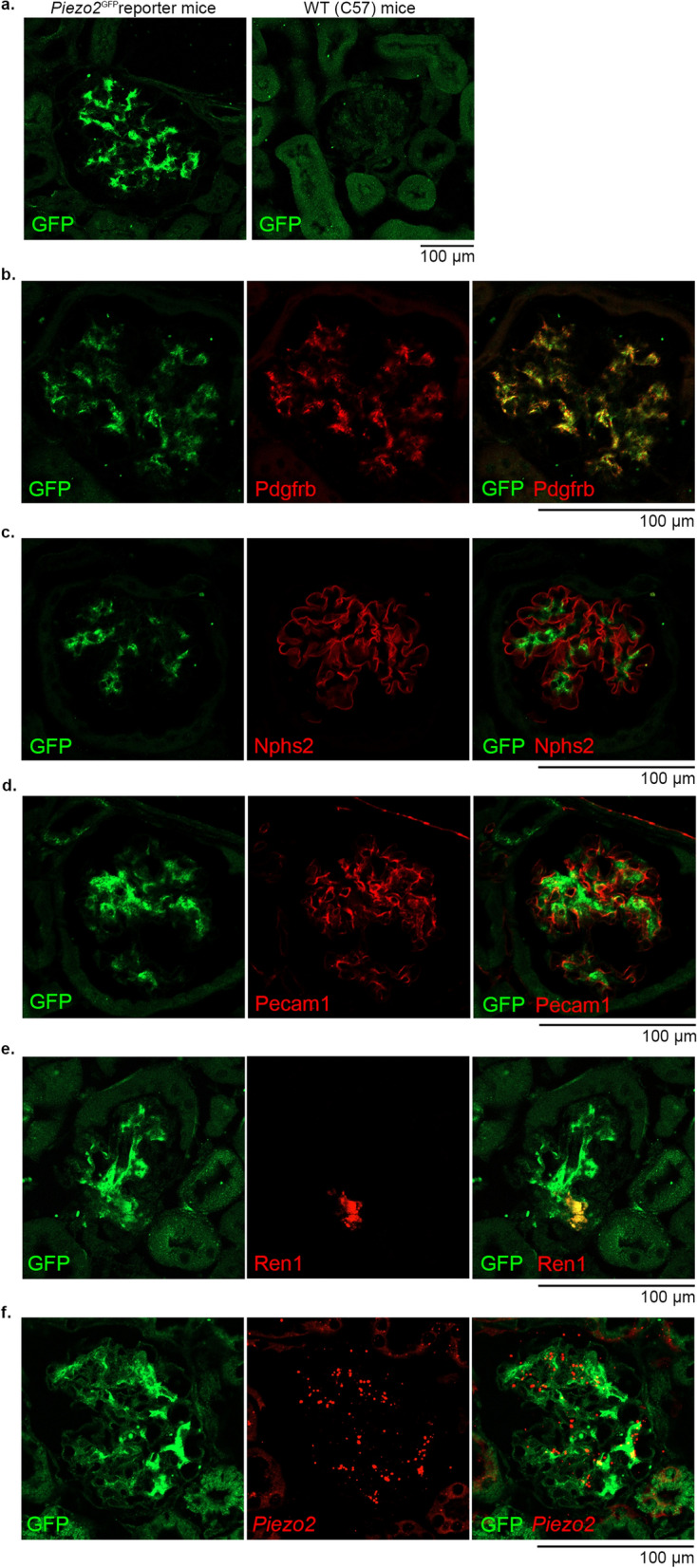
Expression and localization of Piezo2 protein in the kidneys of *Piezo2*-*GFP*-*IRES*-*Cre* (*Piezo2*^*GFP*^) reporter mice. (**a**) Fluorescence immunostaining of GFP (green) representing Piezo2 protein expression in the kidneys of *Piezo2*^*GFP*^ reporter (left) and wild-type (right) mice. Scale bar, 100 µm. (**b**–**e**) Fluorescence double immunostaining of GFP (green) and cell markers (red), Pdgfrb, Nphs2 (a podocyte marker), Pecam1 (an endothelial cell marker), and Ren1. Scale bars, 100 µm. (**f**) GFP immunostaining (green) and RNAscope in situ hybridization of *Piezo2* (red) in the adjacent serial sections. Scale bar, 100 µm.

We next examined whether Piezo2 is expressed in mesangial cells and renin-producing cells at the protein level. Since the commercially available anti-human Piezo2 antibodies did not cross-react with mouse Piezo2 (data not shown), we analyzed the previously described *Piezo2*-*GFP*-*IRES*-*Cre* (*Piezo2*^*GFP*^) reporter mice^23,28^, in which the Piezo2-GFP fusion protein is expressed under the control of the native *Piezo2* promoter. GFP immunofluorescence staining using an anti-GFP antibody revealed distinct signals within the glomeruli (Fig. 2a, left) in a similar pattern to that seen using in situ hybridization, indicating that the overall histological distributions of *Piezo2* transcripts and the Piezo2-GFP protein were the same. No signal was observed in the kidneys of wild-type C57BL/6J mice (Fig. 2a, right). Double immunofluorescence staining revealed that the Piezo2-GFP protein was colocalized with Pdgfrb (Fig. 2b) and Ren1 (Fig. 2e), supporting Piezo2-GFP expression in mesangial cells and renin-producing cells. The Piezo2-GFP fusion protein was not found to be colocalized with the podocyte marker Nphs2 (also known as podocin, Fig. 2c). There was no or only faint co-staining with the endothelial marker platelet/endothelial cell adhesion molecule 1 (Pecam1, Fig. 2d). Although it was technically difficult to perform immunostaining and RNAscope in situ hybridization on the same section, we confirmed that Piezo2-GFP protein and *Piezo2* transcripts were mostly colocalized using adjacent serial sections with 4 µm thickness (Fig. 2f).

We further determined the cellular localization of Piezo2-GFP using immunoelectron microscopy. Positive DAB staining for Piezo2-GFP was observed as osmium black dots under a transmission electron microscope, which was detected in the cytoplasmic membrane of mesangial cells (Supplementary Fig. S2a,b, arrows), and of renin-producing cells (Supplementary Fig. S2c,d, arrows).

Taken together, the analysis of *Piezo2*^*GFP*^ reporter mice showed that Piezo2 is expressed in mesangial cells and renin-producing cells at the protein level.

### *Piezo2* mRNA is developmentally expressed in *Foxd1*-positive stromal progenitor cells and their derivatives in mouse embryos

**Figure 3:**
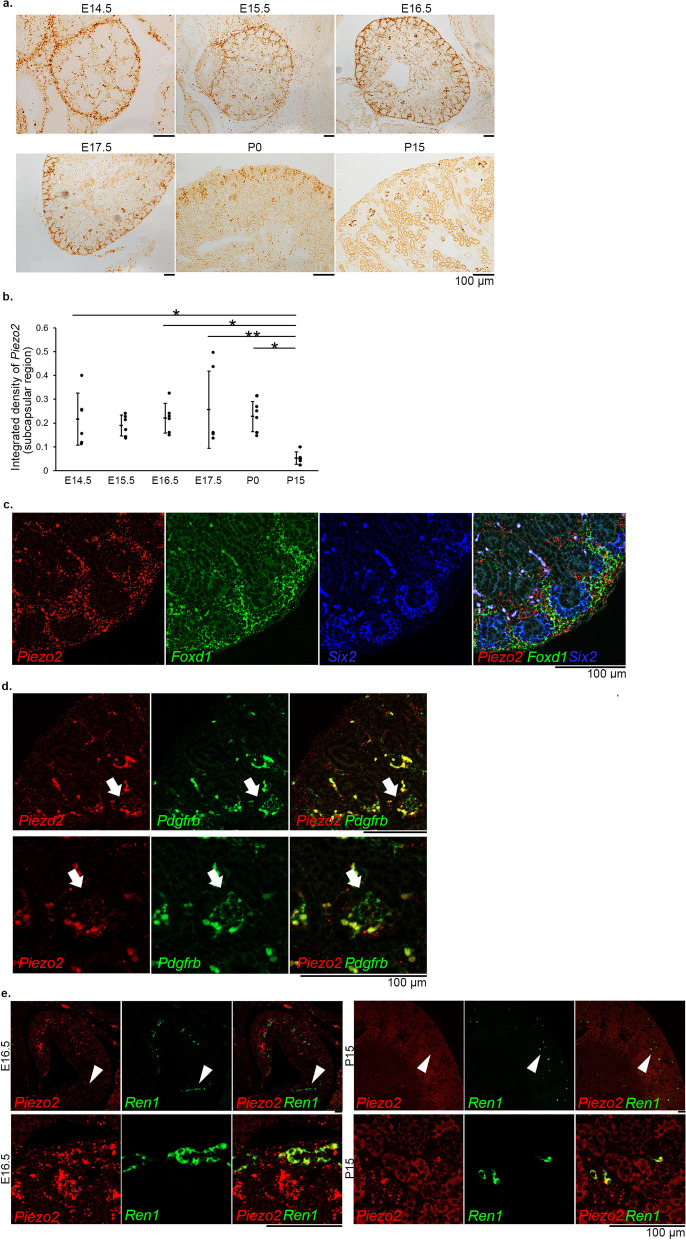
Gene expression and localization of *Piezo2* in the developing mouse kidney. (**a**) RNAscope in situ hybridization of *Piezo2* in the metanephros of embryonic day (E)14.5, E15.5, E16.5, E17.5, and in the kidney of postnatal day (P)0 and P15. Signals were visualized as brown dots by DAB chromogen reaction. (**b**) Quantification of RNAscope in situ hybridization of *Piezo2* in the subcapsular region. *P < 0.05, **P < 0.01 vs. P15, one-way ANOVA followed by Bonferroni's post hoc test (*n* = 6). (**c**) Fluorescence triple RNAscope in situ hybridization of *Piezo2* (red) and cell-lineage markers *Foxd1* (green; a stromal progenitor marker) and *Six2* (blue; a nephron progenitor marker) in the E16.5 metanephros. (**d**) Fluorescence double RNAscope in situ hybridization of *Piezo2* (red) and *Pdgfrb* (green) in the E16.5 metanephros. The lower panels show magnified view of the developing glomeruli (arrows), which contain *Piezo2*-expressing *Pdgfrb*-positive mesangial cells. (**e**) Fluorescence double RNAscope in situ hybridization of *Piezo2* (red) and *Ren1* (green) in the E16.5 metanephros and P15 kidney. The lower panels show magnified view of *Ren1*-expressing cells (arrowheads; left, *Piezo2*-expressing *Ren1*-positive arterioles; right, *Piezo2*-expressing juxtaglomerular *Ren1*-positive cells). Scale bars, 100 µm.

To investigate the embryonic origin of *Piezo2/Pdgfrb*- or *Ren1*-positive cells, we next examined the developmental changes in *Piezo2* gene expression in mouse metanephros from embryonic day (E)14.5 until E17.5 and in the kidneys of postnatal day (P)0 and P15 mice. RNAscope in situ hybridization followed by DAB detection revealed that *Piezo2* mRNA was highly expressed in the subcapsular metanephric mesenchyme, developing glomeruli, and some arteriolar cells (Fig. 3a). Quantitative analysis revealed that *Piezo2* gene expression in the subcapsular region did not change during the embryonic period and at P0, and that the expression significantly decreased at P15 (Fig. 3b), possibly due to the disappearance of the metanephric mesenchyme.

Fluorescence multiple RNAscope in situ hybridization of the E16.5 metanephros revealed that most of the *Piezo2*-expressing cells in the subcapsular metanephric mesenchyme were *Foxd1*-positive stromal progenitor cells and not *Six2*-positive nephron progenitor cells (Fig. 3c). *Piezo2*-expressing cells in the developing glomeruli were positive for the mesangial marker *Pdgfrb* (Fig. 3d). *Ren1* transcripts were observed in the arterioles of the E16.5 kidney, where co-expression of *Piezo2* signals was noted (Fig. 3e). *Ren1* expression was restricted to the juxtaglomerular cells at P15, at which time *Piezo2* mRNA was also expressed.

### *Piezo2* expression is increased in renin-producing cells under dehydration conditions

**Figure 4:**
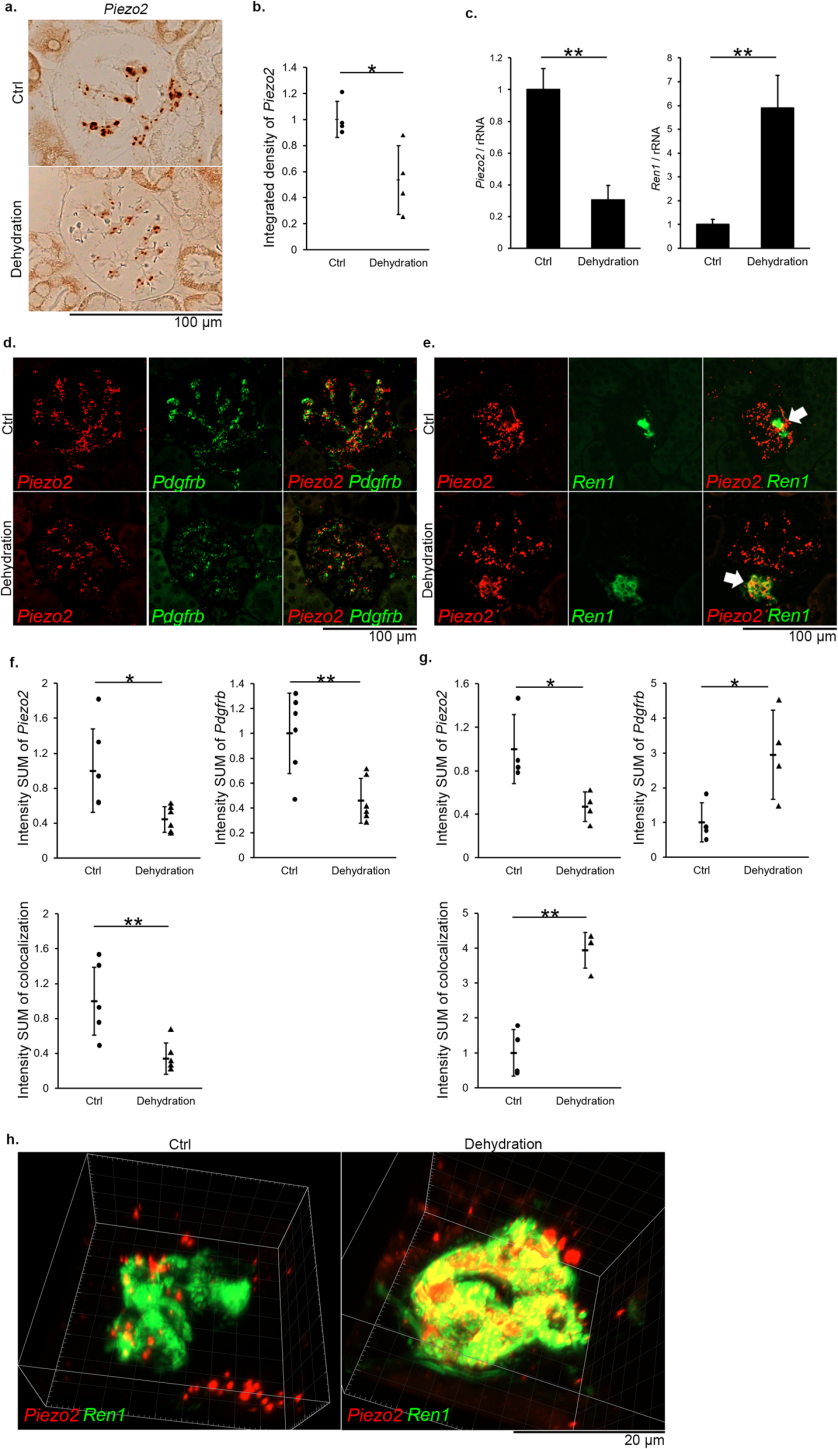
Alteration of *Piezo2* gene expression in a mouse model of dehydration. C57BL/6J mice were fed a normal rodent chow with drinking water supplied ad libitum (Ctrl) or mice were fed a normal rodent chow and deprived of drinking water for 5 days (Dehydration). (**a**) Representative images of RNAscope in situ hybridization of *Piezo2* in the kidneys of Ctrl (*n* = 5) and Dehydration (*n* = 5) mice. Signals were visualized as brown dots by DAB chromogen detection. (**b**) Quantification of RNAscope in situ hybridization of *Piezo2* in the glomeruli of Ctrl and Dehydration mice. *Piezo2* expression in the Ctrl group is arbitrarily expressed as 1. *P < 0.05 vs. Ctrl, a two-tailed unpaired Student’s *t*-test (*n* = 4 per group). (**c**) Quantitative RT-PCR analysis of *Piezo2* (left) and *Ren1* (right) in the kidneys of Ctrl and Dehydration mice. **P < 0.01 vs. Ctrl, a two-tailed unpaired Student’s *t*-test (*n* = 6 per group). (**d**,**e**) Representative fluorescence double RNAscope in situ hybridization of *Piezo2* (red) and *Pdgfrb* (green, **d**) and *Ren1* (green, **e**) in the kidneys of the Ctrl and Dehydration groups. Arrows indicate juxtaglomerular regions. (**f**) Quantification of double RNAscope in situ hybridization of *Piezo2* (upper left) and *Pdgfrb* (upper right) in the glomeruli of the Ctrl and Dehydration groups. The colocalized signal density is shown in the lower panel. *P < 0.05, **P < 0.01 vs. Ctrl, a two-tailed unpaired Student’s *t*-test (*n* = 6 per group). (**g**) Quantification of double RNAscope in situ hybridization of *Piezo2* (upper left) and *Ren1* (upper right) in the glomeruli of the Ctrl and Dehydration groups. The colocalized signal density is shown in the lower panel. *P < 0.05, **P < 0.01 vs. Ctrl, a two-tailed unpaired Student’s *t*-test (*n* = 4 per group). (**h**) Three-dimensional images of double RNAscope in situ hybridization for *Piezo2* (red) and *Ren1* (green) in the juxtaglomerular regions of Ctrl group (left) and Dehydration group (right) (corresponding to arrows in **e**, the boxed areas in Supplementary Fig. S3a,b). Z-stack sections were acquired with a Zeiss LSM980 confocal laser scanning microscope by defining the top and bottom focal plane positions and a step size of 1 µm (total 20 focal planes for Ctrl group, 40 focal planes for Dehydration group). Scale bars, (**a**), (**d**), (**e**), 100 µm; (**h**), 20 µm.

We further used a mouse model of dehydration to investigate whether *Piezo2* expression is altered in response to reduced mechanical strain in the glomeruli and afferent arterioles. RNAscope in situ hybridization followed by DAB detection revealed that overall *Piezo2* dot signals were weakened in the dehydration group (Fig. 4a). Quantitative analysis of signal density indicated that glomerular *Piezo2* expression was significantly decreased in the dehydration group (*P* < 0.05 vs. control group, Fig. 4b). Quantitative RT-PCR also showed that *Piezo2* expression was lower in the dehydration group (*P* < 0.01 vs. control group), whereas *Ren1* was overexpressed in the dehydration group (*P* < 0.01) (Fig. 4c).

Double RNAscope in situ hybridization showed that *Piezo2* signals in *Pdgfrb*-positive mesangial cells were diminished by dehydration (Fig. 4d). Notably, *Ren1* expression was strongly augmented in the juxtaglomerular area in the dehydration group, where *Piezo2* expression was also enhanced (Fig. 4e). Quantitative analysis revealed that both *Piezo2* and *Pdgfrb* expression decreased in the dehydration group (*P* < 0.05 and *P* < 0.01 vs. control group, Fig. 4f, upper panels). The colocalized signal density of *Pdgfrb* and *Piezo2* was also significantly reduced in the dehydration group (*P* < 0.01, Fig. 4f, lower panel). For *Piezo2* and *Ren1* double staining, although *Piezo2* expression was again decreased by dehydration (*P* < 0.05, Fig. 4g, upper left panel), *Ren1* expression and the colocalized density of *Ren1* and *Piezo2* were enhanced in the dehydration group (*P* < 0.05 and *P* < 0.01, respectively, Fig. 4g, upper right and lower panels).

There may be a concern of whether the colocalization of *Piezo2* and *Ren1* can be evaluated accurately in a plan view because various cells are densely packed in the juxtaglomerular region. Thus, to clarify their co-expression in more detail, we created 3D images of *Ren1*-expressing cells using the Z-stack system of the confocal laser scanning microscope. When viewed from different directions by rotating the images, *Piezo2* transcripts were definitely co-expressed in *Ren1*-positive cells in the control group (Fig. 4h, left, Supplementary Video S1 and Supplementary Fig. S3). Additionally, we detected a few *Piezo2-*positive cells outside the *Ren1*-positive area*.* These cells were located in the space between the *Ren1*- or *Acta2*- positive arterioles and renal tubules in the vascular pole, suggesting that they are extraglomerular mesangial cells (Supplementary Fig. S4). Notably, the juxtaglomerular *Ren1*-positive area was markedly enlarged in the dehydration group where *Piezo2* and *Ren1* transcripts were definitely colocalized (Fig. 4h, right, Supplementary Video S2 and Supplementary Fig. S3). The 3D reconstruction data strengthen our conclusion that *Piezo2* is expressed in *Ren1*-positive cells.

We further used *Piezo2*^*GFP*^ reporter mice to confirm the above-mentioned results of the dehydration model. Glomerular GFP staining was enhanced in the dehydration group compared with that in the control group (Supplementary Fig. S5a, left, middle). No GFP signal was detected in the kidneys of wild-type C57BL/6J mice (Supplementary Fig. S5a, right). Double immunofluorescence revealed that Piezo2-GFP protein and mesangial Pdgfrb signals in the glomeruli were lower in the dehydration group than in the control group (Supplementary Fig. S5b). By contrast, Ren1 staining was augmented, and colocalization of Piezo2-GFP and Ren1 was increased in the dehydration group (Supplementary Fig. S5c), recapitulating the results of RNAscope analyses (Fig. 4).

### *Piezo2* gene knockdown and mechanical stretch suppress *Ren1* gene expression in cultured mouse renin-producing cell line As4.1

**Figure 5:**
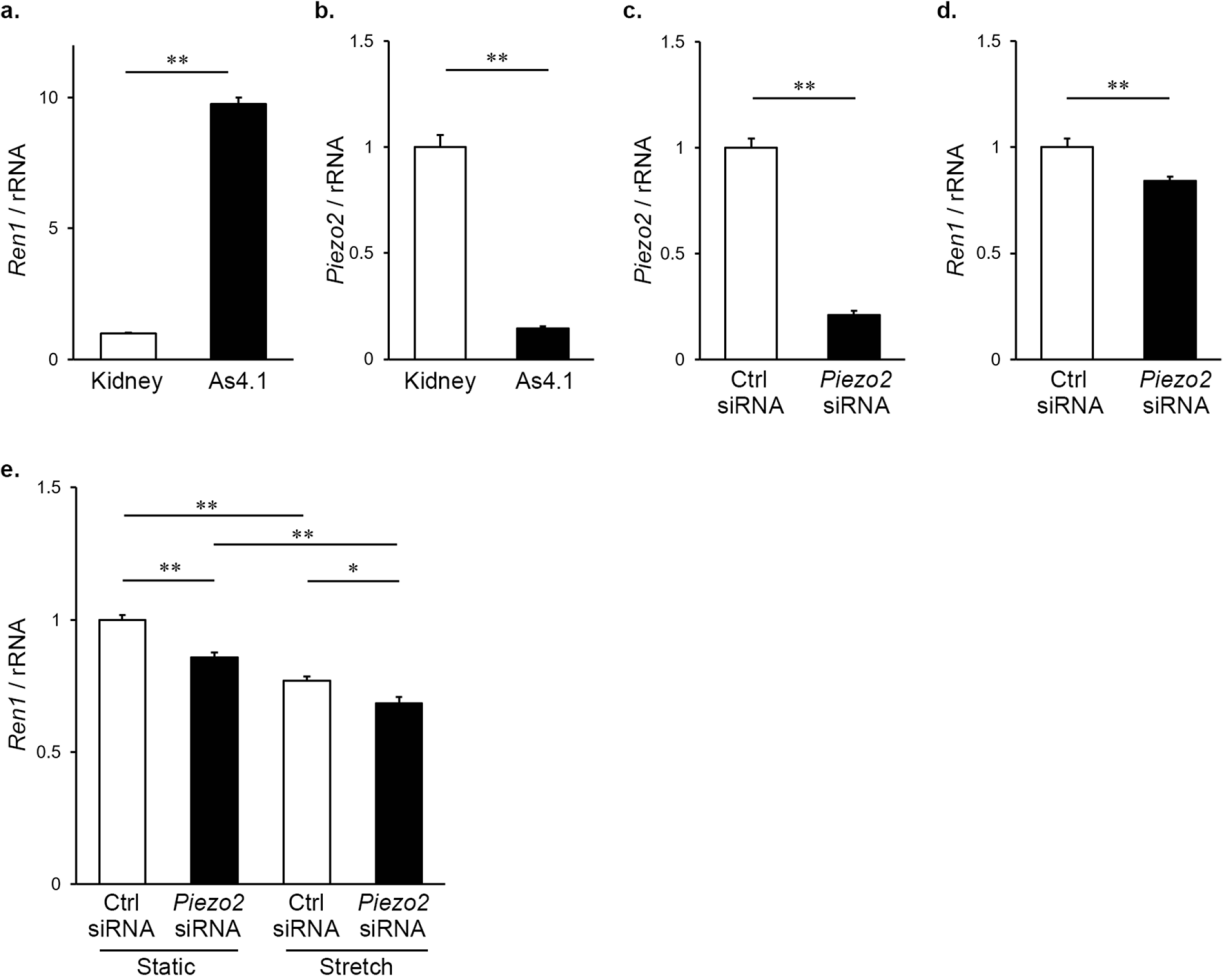
Inhibition of *Ren1* expression by *Piezo2* knockdown and mechanical stretch in cultured renin-producing As4.1 cell line. (**a**) Quantitative RT-PCR analysis of *Ren1* in the mouse kidney and As4.1 cells. *Ren1* expression in the kidney is arbitrarily expressed as 1. **P < 0.01 vs. kidney, a two-tailed unpaired Student’s *t*-test (*n* = 5). (**b**) Quantitative RT-PCR analysis of *Piezo2* in the kidney and As4.1 cells. *Ren1* expression in the kidney is arbitrarily expressed as 1. **P < 0.01 vs. kidney, a two-tailed unpaired Student’s *t*-test (*n* = 5). (**c**) Quantitative RT-PCR analysis of *Piezo2* in As4.1 cells transfected with control siRNA and *Piezo2* siRNA. *Piezo2* expression in cells transfected with control siRNA is arbitrarily expressed as 1. **P < 0.01 vs. control siRNA group, a two-tailed unpaired Student’s *t*-test (*n* = 9). (**d**) Quantitative RT-PCR analysis of *Ren1* in As4.1 cells transfected with control siRNA and *Piezo2* siRNA. *Ren1* expression in cells transfected with control siRNA is arbitrarily expressed as 1. **P < 0.01 vs. control siRNA group, a two-tailed unpaired Student’s *t*-test (*n* = 9). (**e**) Quantitative RT-PCR analysis of *Ren1* in As4.1 cells transfected with control siRNA and *Piezo2* siRNA under static and stretched conditions. *Ren1* expression in control siRNA-transfected cells under static condition is arbitrarily expressed as 1. Statistical analysis was performed by two-way ANOVA, *P* < 0.01 knockdown effect, *P* < 0.01 stretch effect, not significant interaction effect. **P < 0.01, *P < 0.05 by Bonferroni's post hoc test (*n* = 9 per group).

Finally, we performed in vitro experiments using the cultured mouse renin-producing cell line As4.1 and investigated the effects of *Piezo2* knockdown and mechanical load on *Ren1* mRNA expression. We confirmed that As4.1 cells abundantly expressed *Ren1* mRNA compared with the whole kidney sample (Fig. 5a). The cells also expressed *Piezo2* mRNA, although the level was lower than that in the whole kidney (Fig. 5b).

*Piezo2* mRNA knockdown was conducted in As4.1 cells with three types of *Piezo2* siRNA and control siRNA, using Lipofectamine™ RNAiMAX transfection reagent. After 72 h of transfection, only one *Piezo2* siRNA could sufficiently inhibit *Piezo2* mRNA expression (79% reduction; *P* < 0.01 vs. control siRNA) (Fig. 5c). We used this construct in the following experiments. The siRNA-mediated specific knockdown of the *Piezo2* gene partially but significantly reduced *Ren1* mRNA expression (Fig. 5d).

It was previously reported that cyclic mechanical distension suppresses renin gene transcription in As4.1 cells^35^. Therefore, we next examined the effects of *Piezo2* knockdown and mechanical stretch. Cells were seeded onto silicon stretch chambers coated with collagen I. After transfection of control siRNA and *Piezo2* siRNA for 72 h, the cells were exposed to uniaxial cyclic tensile strain (12% elongation, 0.5 Hz) for 24 h. Static cells were plated onto the same chambers, treated with control or *Piezo2* siRNA, and were not subjected to cyclic stretch.

As shown in Fig. 5e, *Piezo2* knockdown by siRNA lowered *Ren1* gene expression under both static and stretched conditions. Cyclic mechanical strain also decreased *Ren1* mRNA expression in both control siRNA and *Piezo2* siRNA groups. There were statistical significant knockdown effect (*P* < 0.01) and stretch effect (*P* < 0.01), while there was no interaction effect (two-way ANOVA, *n* = 9 per group). The post hoc test revealed that *Piezo2* knockdown significantly reduced *Ren1* expression in both static (*P* < 0.01) and stretched (*P* < 0.05) conditions (Fig. 5e).

## Discussion

In the present study, we demonstrated that *Piezo2* is expressed in mesangial cells, juxtaglomerular renin-producing cells, and embryonic precursor cells in the mouse kidney. The in vivo alteration of renal *Piezo2* expression was observed under dehydration. Furthermore, *Ren1* expression in cultured As4.1 cells was downregulated by *Piezo2* knockdown, suggesting an indispensable role for Piezo2 in body fluid balance.

We used RNAscope in situ hybridization technology to visualize *Piezo2* transcripts and cell marker genes. This method was successfully utilized in a previous study by Zeng et al.^27^ to detect *Piezo2* expression in the nodose ganglion. Compared with the conventional in situ hybridization techniques, RNAscope has high sensitivity and specificity and enables multiplex analysis with fluorescent dyes^32^. We could not obtain satisfactory results from Piezo2 immunostaining using commercially available antibodies (data not shown); thus, we further used *Piezo2*^*GFP*^ reporter mice to assess the intrarenal distribution of the Piezo2-GFP fusion protein using anti-GFP immunostaining, as described previously^23,28^ (Fig. 2a). The expression patterns of *Piezo2* transcripts and the Piezo2-GFP fusion protein were similar. Furthermore, we performed immunofluorescence staining of the Piezo2-GFP protein and in situ hybridization of *Piezo2* mRNA in serial sections to confirm their similar expression pattern (Fig. 2f). Since it was difficult to perform double staining of immunofluorescence and in situ hybridization on the same section, their consistency was verified using adjacent sections.

We identified clear Piezo2 gene and protein expression in Pdgfrb-positive mesangial cells in normal mouse kidneys. Mesangial cells have pivotal mechanosensitive roles in integrating the morphology of the glomerular tuft against capillary tension^5,6^. Indeed, cultured mesangial cells, under mechanical stimuli such as stretching, upregulate growth factors, including transforming growth factor beta 1 (*Tgfb1*)^9^, vascular endothelial growth factor A (*Vegfa*)^36^ and cellular communication network factor 2 (*Ccn2*, also known as connective tissue growth factor)^37^, thereby promoting fibrotic change and ECM deposition^7,8^. Mechanosensors in mesangial cells remained unidentified for a long time. Quite recently, Fu et al.^38^ reported that *Piezo1* is involved in ECM gene expression in cultured mesangial cells under mechanical stress and in renal fibrosis in a mouse model. They used an unconventional method of culturing cells on hydrogel with different stiffness to evaluate the effects of mechanical stress. They described that the stiffer gels induced more ECM upregulation, which was ameliorated by inhibition of Piezo1 and related signaling molecules. However, Piezo2 was not mentioned in their paper. Data from our present study suggest the possibility that Piezo2 may also contribute to the mechanosensing reactions of mesangial cells, although further studies are necessary to confirm this.

We observed Piezo2 gene and protein expression in Ren1-positive cells in the juxtaglomerular apparatus. Seghers et al.^13^ examined which of various candidate mechanosensitive ion channels mediate renin secretion from the renin-expressing As4.1 cells. They considered that *Piezo2* mRNA expression was low, although present, in the cell line, and a detailed analysis was not performed. They demonstrated that knockdown of *Trpv4*, but not *Trpv2* or *Piezo1*, inhibited the calcium (Ca) response to mechanical strain in the cell line. They further performed an in vivo analysis showing that Trpv4 is expressed in the juxtaglomerular cells and proximal tubules, and that systemic knockout of *Trpv4* resulted in higher plasma renin levels. Our data in normal and reporter gene expressing mice, as well as in the dehydration model and in vitro analysis using the same As4.1 cells as discussed below, suggest that Piezo2 may also modulate renin release as another mechanosensor.

Our findings on Piezo2 expression in mesangial cells and renin-producing cells may further be supported by recent single-cell transcriptome analyses of mouse glomerular/mesangial cells. For example, He et al.^39^ performed single-cell RNA sequencing of mouse glomerular cells and detected heterogeneity in glomerulus-associated Pdgfrb-expressing cells. There were 4 distinct subpopulations, including *bona fide* intraglomerular mesangial cells, renin-producing cells, vascular smooth muscle cells, and extraglomerular mesangial cells. It was found that *Piezo2* was mainly expressed in intraglomerular mesangial cells, but also in renin-producing cells at lower levels, confirming our results. Karaiskos et al.^40^ reported a single-cell transcriptome atlas of the mouse glomerulus, and *Piezo2* expression was found in the mesangial and glomerular endothelial populations by searching their database (https://shiny.mdc-berlin.de/mgsca/). Lu et al.^41^ also performed single cell RNA-sequencing analysis using 14 mouse glomerular mesangial cell samples, and *Piezo2* as well as *Trpv2* and *P2rx1* was included in the list of mesangial cell essential genes. Our present study suggested that Piezo2 was additionally expressed at lower levels in glomerular endothelial cells and extraglomerular cells, and these findings may not be inconsistent with the above-mentioned single cell RNA analyses.

*Piezo2* was found to be expressed in subcapsular *Foxd1*-positive cells but not in *Six2*-positive cells in the developing metanephros. In mice, the kidney develops from the intermediate mesoderm-derived metanephric mesenchyme and ureteric bud through reciprocal inductive interactions. The ureteric bud forms the collecting ducts, the renal pelvis, and the ureter^16^. The cap mesenchyme around the ureteric bud tips contains *Six2*-positive nephron progenitor cells, which differentiate into podocytes, parietal epithelial cells of Bowman’s capsule, proximal tubules, the loop of Henle, distal tubules, and connecting tubules^17^. The loose mesenchyme around the cap mesenchyme gives rise to *Foxd1*-positive stromal cells, which in turn give rise to the glomerular mesangial cells and mural cells of the renal arterial tree (renin cells, smooth muscle cells, perivascular fibroblasts, and pericytes)^14,15,18^. The expression of Piezo2 in mesangial cells and juxtaglomerular renin-producing cells, but not in the proximal and distal tubules and collecting ducts, is in agreement with the embryonic origin of *Foxd1*-positive cells. *Piezo2* mRNA was expressed in *Pdgfrb*-positive glomerular cells and *Ren1*-positive cells in the embryo. Subcapsular *Piezo2* expression was parallel to the presence of metanephric mesenchyme. Nephrogenesis is not completed at birth; the metanephric mesenchyme disappears at around P3^42^. Consequently, *Piezo2* mRNA expression in the subcapsular metanephric mesenchyme persisted at P0 but disappeared at P15. The functions of Piezo2 in embryonic *Foxd1-*positive cells should be analyzed in the future.

In the mouse model of dehydration, *Piezo2* expression in the whole kidney was reduced. This result may be attributed to the decrease in transcript levels in mesangial cells. The number of mesangial cells does not seem to decrease; thus, *Piezo2* expression itself may be directly downregulated, presumably by intraglomerular hypotension. Conversely, the signal intensity of *Piezo2* expression in juxtaglomerular renin-producing cells was markedly upregulated. It is noteworthy that the area of renin-producing cells was enlarged. It is known that renin-producing cells have lineage plasticity; when body fluid homeostasis is threatened and demand for renin release is increased, vascular smooth muscle cells adjacent to the juxtaglomerular area acquire the phenotype of renin-producing cells and secrete renin^12,43^. It is tempting to speculate that such transdifferentiation might be triggered by mechanotransduction through Piezo2.

In general, an increase in Ca influx promotes exocytosis in secretory cells. On the other hand, renin release from juxtaglomerular cells is suppressed by Ca influx, a phenomenon known as calcium paradox of renin secretion^13^. The increased renin expression in our dehydration model should accompany decreased Ca, and thus Piezo2 inactivation. However, our results revealed Piezo2 upregulation in the dehydration model. We consider that these seeming contradictory Ca changes might be explained by the dual- or triple-feedback control system for the homeostasis of intracellular pressure. It is known that different types of mechanosensitive channels mediating different roles are localized on the same cells. For example, Du et al.^44^ reported that Trpv4, Piezo1, and Piezo2 are expressed on the same chondrocyte, each of which transduces mechanical stimuli differentially, depending on the stimulus intensities. Nakazawa et al.^45^ reported that Trpv1 and Trpv4 are expressed in lens surface cells. Trpv1 senses decreased pressure or hyperosmotic stress, whereas Trpv4 senses increased pressure or hypoosmotic stress. These reciprocal stimuli are transduced through distinct signaling pathways according to Trpv1/4, thereby forming dual-feedback systems controlling intracellular pressure or osmolarity. In such systems, there may be a possibility that reciprocal Ca changes might lead to unexpected observations. Indeed, Trpv4 is reported to be expressed in juxtaglomerular cells and As4.1 cells^13^, suggesting the possibility that the above-mentioned complicated crosstalk of Ca balance exists in juxtaglomerular cells. Further research will be needed to address this point in the future.

For evaluation of Piezo2 functions, in vivo analysis using *Piezo2* knockout mice will be ideal. However, systemic *Piezo2* knockout mice are lethal at P1. Generation of conditional renin-cell specific knockout mice using Cre recombinase will be rather time-consuming. Thus, in this study, we decided to perform in vitro analysis using As4.1 cells. It was revealed that *Piezo2* knockdown resulted in the downregulation of *Ren1* expression in both the static and stretched conditions. These findings suggest that Piezo2 functions upstream of Ren1 signaling and positively regulates its expression. Although this positive regulation is again contradictory to the “calcium paradox”, our in vitro data are in agreement with our in vivo finding of reduced mechanical stress and increased *Ren1* expression in the dehydration model. It should also be noted that the stretch-induced reduction of *Ren1* expression was not rescued by *Piezo2* knockdown. This result suggests the possibility that the effect of mechanical stretch in our experimental condition was not mainly transduced by the Piezo2 channel. As discussed above, different mechanosensors in the same cell occasionally mediate different modes of mechanical stimuli, such that Trpv1 and Trpv4 on lens cells sense decreased and increased pressure, respectively. Thus, it should be better to evaluate mechanotransduction of Piezo2 in the context of multimodal mechanical stimuli, including osmotic stress, increased or decreased hydrostatic pressure, and deformation. Further sophistication of the experimental procedures will be necessary in the analysis of mechanical stress.

Our study has several limitations. Our morphological study suggests that *Piezo2* is also expressed at low levels in the glomerular endothelial cells and extraglomerular mesangial cells. However, the data are not so convincing. If a good Piezo2 antibody becomes available in the future, it will be possible to identify cells and intracellular localization sites more clearly. Further functional analyses of Piezo2 ares also required. For example, electrophysiological analysis of the Piezo2 channel will clarify how the Ca change against the calcium paradox works. Investigation of molecular pathways upstream and downstream of Piezo2 will be interesting, considering the possible crosstalk of signaling cascades of other mechanosensors, including Piezo1 and Trpv4. In vivo Piezo2-knockout experiments will be very important to really substantiate the indispensability of Piezo2 in body fluid control.

In conclusion, the present study clearly revealed the expression of *Piezo2* in the developing and adult mouse kidneys in normal and dehydrated conditions. We also showed that Piezo2 functions upstream of Ren1 in vitro. Our study suggests crucial roles for Piezo2 in renal morphology and physiology. This work may be an initial step toward clinical understanding and intervention of mechanical stress-related kidney diseases, such as hypertensive and diabetic nephropathy.

## Methods

### Ethical statement

The research protocols were approved by the Animal Research Committee of Kyorin University (Permit Number: Animal 216) and Recombinant DNA Experiments Safety Committee of Kyorin University (Permit Number: 179-2). All animal experiments were performed in strict accordance with the institutional guidelines and the recommendations in the ARRIVE guidelines^46^.

### Mice

Male C57BL/6J mice (6 to 8-week-old) and female pregnant C57BL/6J mice carrying E11.5–E15.5 embryos were purchased from Sankyo Labo Service Corporation (Tokyo, Japan). *Piezo2*-*GFP*-*IRES*-*Cre* (*Piezo2*^*GFP*^) reporter mice were previously described^23,28^.

For developmental analysis, E14.5–E17.5 mouse embryos were dissected out from pregnant mice under isoflurane anesthesia. Whole embryos (E14.5–E16.5), isolated metanephros (E17.5), and kidneys (P0 and P15) were fixed in 4% paraformaldehyde overnight at 4 °C, and paraffin sections (5 μm) were prepared.

To establish the in vivo model of reduced mechanical load to the glomeruli and juxtaglomerular renin-producing cells, 8-week-old male C57BL/6J mice were divided into a control group fed a standard rodent chow and drinking water ad libitum and a dehydration group fed a standard rodent chow and deprived of drinking water for 5 days (*n* = 6 for each). According to previously reported methods^47,48^, we initially performed 1, 3, 4, and 5 days of dehydration as preliminary experiments. Then, we determined a duration of 5 days for the final experiment. The same control and dehydration interventions were also given to *Piezo2*^*GFP*^ mice (*n* = 2 for each).

Mice were housed in a room maintained at constant temperature (20 to 26 °C), humidity (40 to 60%), and light cycle (12-h light/dark). The health condition of the mice was checked every day. Mice were anesthetized with isoflurane, and all efforts were made to minimize suffering.

### RNAscope in situ hybridization

RNAscope in situ hybridization was performed using an RNAscope 2.5 HD detection kit (brown), RNAscope multiplex fluorescent reagent kit v2, commercially available paired double-Z oligonucleotide probes (Advanced Cell Diagnostics, Newark, CA, USA), and a TSA plus system (Akoya Biosciences, Marlborough, MA, USA)^32^. The target probes used are: Mm-*Piezo2*-C1, Mm-*Piezo2*-C2, Mm-*Pdgfrb*-C2, Mm-*Nphs1*-C2, Mm-*Tek*-C1, Mm-*Ren1*-C1, Mm-*Foxd1*-C3, Mm-*Six2*-C1, negative control bacterial *dapB*-C1, and positive control Mm-*Ppib*-C1. Briefly, mice were perfusion fixed with 4% paraformaldehyde via the left ventricular apex. The kidneys were excised and immersed in the same fixative for 24 h. Paraffin sections (5-μm-thick) were baked for 1 h at 60 °C, deparaffinized, subjected to hydrogen peroxide for 10 min, boiled in target retrieval buffer for 15 min, and treated with protease plus or protease III for 30 min at 40 °C. Cryosections (8 μm) were baked for 30 min at 60 °C, postfixed in 4% paraformaldehyde for 15 min at 4 °C before hydrogen peroxide treatment. The sections were then hybridized with the target probe(s) for 2 h at 40 °C, followed by signal amplification reactions and DAB chromogen detection or fluorescence (Cy3, Fluorescein, Cy5) detection, according to the manufacturer’s protocols. Brightfield images were acquired using a light microscope (BX51; Olympus, Tokyo, Japan). Fluorescence signals were observed using a confocal laser scanning microscope (LSM 980; Zeiss, Oberkochen, Germany). Signal intensities were compared under the same acquisition conditions. Three-dimensional reconstruction was performed using the Z-stack analysis of LSM 980 and Imaris software 9.6 (Bitplane, Oxford Instruments, Belfast, UK).

### Quantitative analysis of RNAscope in situ hybridization

Brightfield images of DAB reaction were analyzed by ImageJ software according to the method by Jensen^49^. The subcapsular region in the embryonic metanephros was selected as the region of interest (ROI) using a rectangular tool. The whole glomerulus in the adult kidney was also selected as ROI. The DAB-mediated brown signals were measured as the integrated density after identical threshold adjustment and compared among groups.

Fluorescent images were analyzed by the Imaris software according to the manufacturer’s instruction. The glomerulus, including the juxtaglomerular apparatus, was selected as ROI, a red or green channel was selected, and the intensity sum was measured after identical threshold adjustment using Imaris Surface command. Colocalization of different colors was measured as colocalized signal density using Imaris Coloc command.

### Gene expression analysis

Kidneys were harvested under isoflurane anesthesia, snap frozen in liquid nitrogen, and stored at − 80 °C. Total RNA was extracted using an RNeasy mini kit (Qiagen, Hilden, Germany), and reverse-transcribed to cDNA with high capacity cDNA reverse transcription kit (Applied Biosystems, Waltham, MA). Gene expression was quantitated by real-time quantitative RT-PCR using StepOnePlus (Applied Biosystems), as previously described^50^. We used TaqMan gene expression assays reagent and commercially available primers and probe sets.

### Isolation of glomeruli

Glomerular fraction was isolated using Dynabeads method^51^. Briefly, mice were perfused with magnetic Dynabeads M-450 (4.5 μm, Dynal, 8 × 10^7^/mouse). Renal cortices were minced, digested in Hank’s balanced salt solution (Thermo Fisher Scientific, Waltham, MA) containing collagenase A (1 mg/ml, Roche Diagnostics, Basel, Switzerland) and DNase I (100 U/ml) for 1 h at 37 °C with gentle agitation. The digested samples were filtered through 100 μm cell strainer. Magnetic beads were trapped in the glomerular capillary tufts, accordingly, glomeruli were separated from nonglomerular cells and connective tissues using a magnet.

### Immunofluorescence staining

For GFP immunostaining, 4% paraformaldehyde-fixed renal cryosections (8 μm) from *Piezo2*^*GFP*^ mice and wild-type mice were pretreated with proteinase type XIV (0.02–0.05%, Sigma, Tokyo, Japan) for 10 min, incubated with 0.5% blocking reagent (Roche Diagnostics), then incubated with a chicken anti-GFP antibody (A10262, 1:100; Thermo Fisher Scientific) overnight at 4 °C, and finally incubated with an Alexa Fluor 488-conjugated anti-chicken IgY (H + L) secondary antibody (A11039, 1:200; Thermo Fisher Scientific)^23,28^. For double immunostaining, GFP-stained sections were incubated with a rabbit anti-Pdgfrb (3169S, 1:100; Cell Signaling Technology, Danvers, MA, USA), rabbit anti-Nphs2 (29040, 1:100; Immuno-Biological Laboratories, Gunma, Japan), rat anti-Pecam1 (553370, 1:200; BD Biosciences, Franklin Lakes, New Jersey, USA), or rabbit anti-Ren1 (ab212197, 1:1000; Abcam, Cambridge, UK), followed by an Alexa Fluor Plus 555-conjugated anti-rabbit IgG (H + L) secondary antibody (A32794, 1:200; Thermo Fisher Scientific) or Cy3-conjugated Affini Pure F(ab′)_2_ fragment anti-rat IgG (H + L) secondary antibody (712-166-153, 1:200; Jackson ImmunoResearch, West Grove, PA, USA). Fluorescence signals were observed using confocal laser scanning microscope (LSM 980; Zeiss).

### Immunoelectron microscopy

4% paraformaldehyde-fixed cryosections (10 µm) were treated with 3% hydrogen peroxide, 0.025% proteinase type XIV for 10 min, avidin–biotin blocking solution, 0.5% blocking reagent, incubated with a chicken anti-GFP antibody overnight at 4 °C, and subsequently with a biotin-conjugated anti-chicken IgY (H + L) secondary antibody (703-066-155, 1:200; Jackson ImmunoResearch). Immunosignals were detected using a Vectastain Elite ABC kit (Vector Laboratories, Burlingame, CA) and a metal-enhanced DAB kit (Thermo Fisher Scientific). Sections were postfixed in 2.5% glutaraldehyde, immersed in 1% OsO_4_, dehydrated, and embedded in Epon 812. Ultrathin sections were stained with uranyl acetate and lead citrate, and observed using JEM-1010 transmission electron microscope (JEOL, Tokyo, Japan)^52^.

### Cell culture

Mouse renin-producing cell line As4.1 was purchased from ATCC (CRL-2193). The cells were maintained in DMEM with high glucose (25 mM) and pyruvate (11995, Thermo Fisher Scientific) supplemented with 10% heat-inactivated FBS (HyClone), penicillin, and streptomycin in a humidified incubator at 37 °C in 5% CO_2_/95% air.

### siRNA transfection

A set of pre-designed Stealth RNAi™ siRNA for mouse *Piezo2* (catalogue No. 1320003 containing 3 siRNA oligonucleotides, ID: MSS212843, MSS212844, and MSS212845) and control siRNA were purchased from Thermo Fisher Scientific, which guarantees high specificity and less unwanted off-target effects. The day before transfection, the cells were seeded on a 6-well plate or stretch chamber (10 cm^2^). Transfection was carried out with siRNA using Lipofectamine RNAiMAX (Themo Fisher Scientific), according to the manufacturer’s instruction. Knockdown efficacy was evaluated by quantitative RT-PCR.

### Cyclic mechanical stretch

As4.1 cells were seeded onto silicon stretch chambers (STB-CH-10, STREX, Osaka, Japan) coated with collagen I (Koken). At 72 h after transfection, a uniaxial cyclic tensile strain (12% elongation, 0.5 Hz) was applied using a cell stretching system (STB-140, STREX) by which a uniform strain can be loaded to the cells along the stretch axis^53^. The stretch frequency and ratio were determined, according to a previous stretch experiment performed on this cell line^35^. Control cells were plated onto the same chambers, and were not subjected to cyclic stretch.

### Statistical analysis

Data are expressed as mean ± s.e.m. Statistical analysis was performed by a two-tailed unpaired Student’s *t*-test for comparison between the two groups, and by one-way or two-way ANOVA followed by Bonferroni's post hoc test for multiple comparisons. Statistical significance was set at *P* < 0.05.

## Supplementary Information

Supplementary Figure S1.

Supplementary Figure S2.

Supplementary Figure S3.

Supplementary Figure S4.

Supplementary Figure S5.

Supplementary Information.

Supplementary Video 1.

Supplementary Video 2.
