# Supplementary figures and images for: Piezo2 expression and its alteration by mechanical forces in mouse mesangial cells and renin-producing cells
Source: Sci Rep. 2022 Mar 10;12:4197. doi: 10.1038/s41598-022-07987-7 (PMC8913706; doi:10.1038/s41598-022-07987-7)

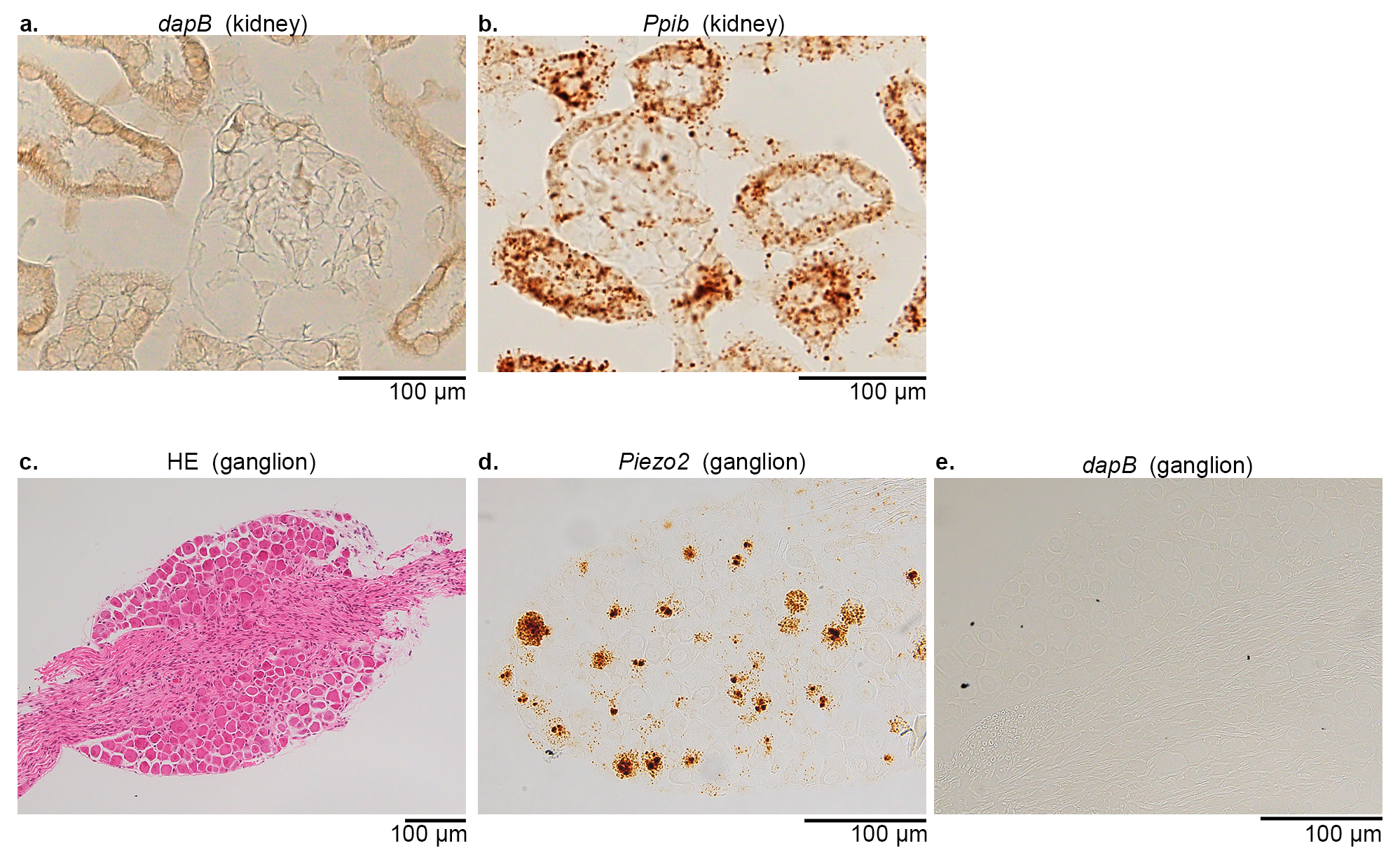

Supplement: Supplementary file 1 — Supplementary Figure S1. [file 41598_2022_7987_MOESM1_ESM.tif]

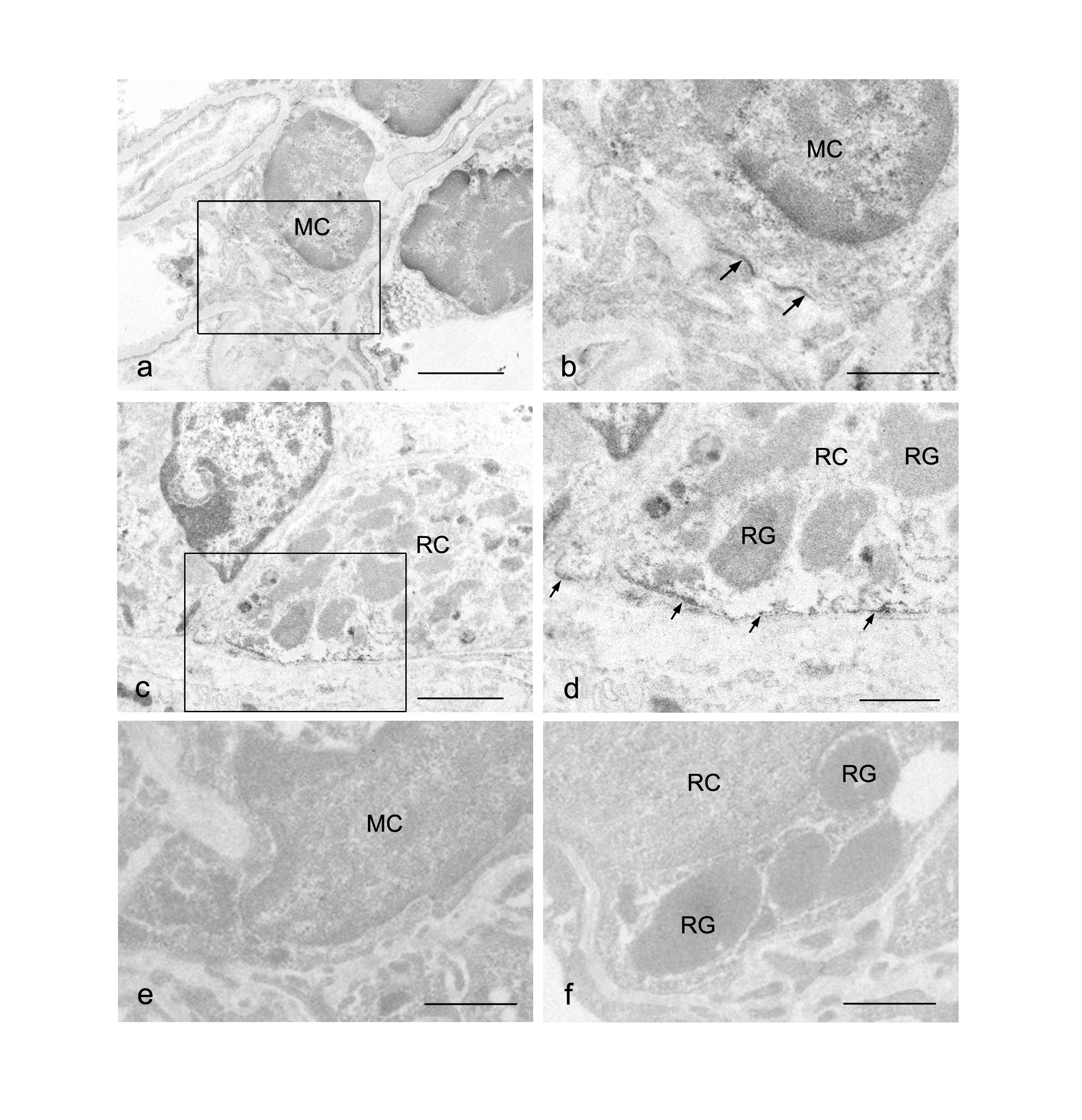

Supplement: Supplementary file 2 — Supplementary Figure S2. [file 41598_2022_7987_MOESM2_ESM.tif]

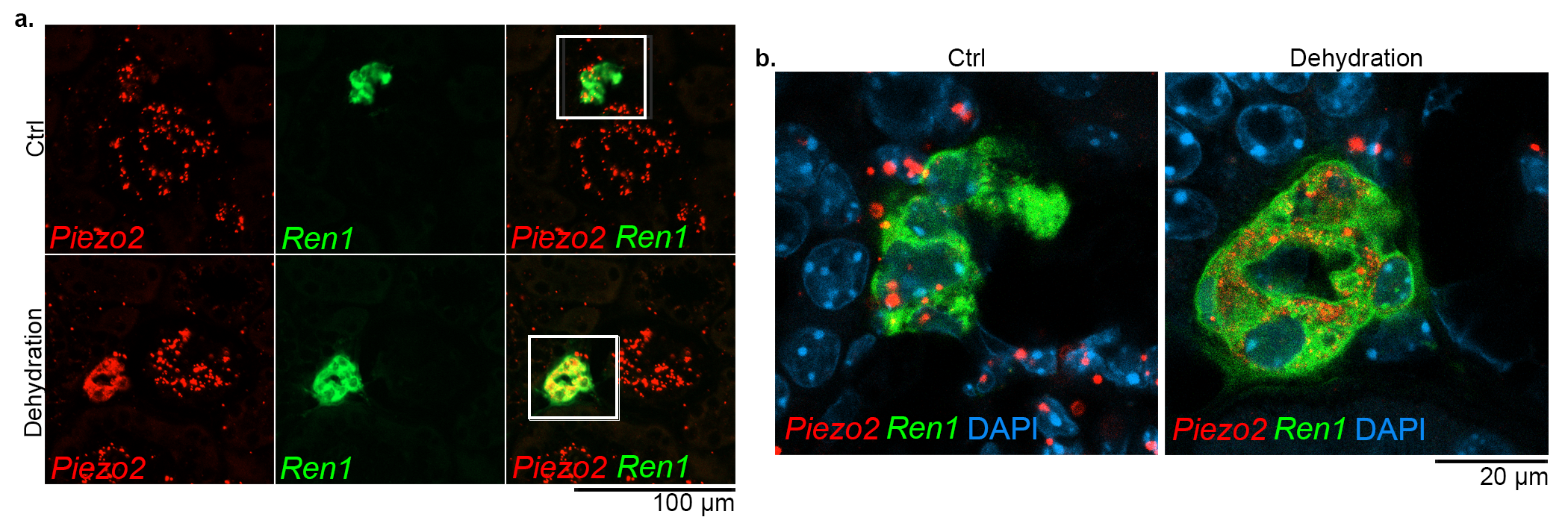

Supplement: Supplementary file 3 — Supplementary Figure S3. [file 41598_2022_7987_MOESM3_ESM.tif]

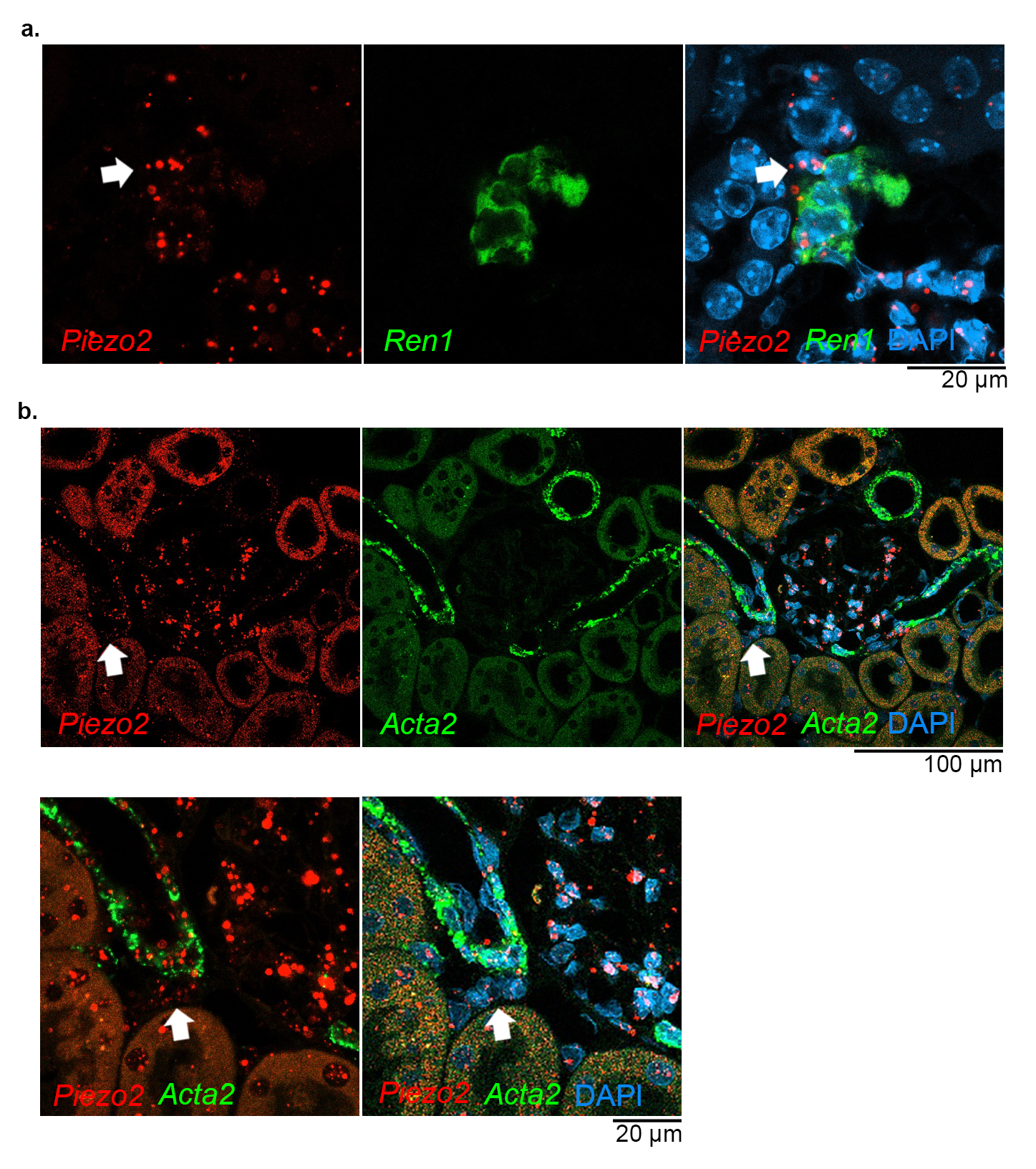

Supplement: Supplementary file 4 — Supplementary Figure S4. [file 41598_2022_7987_MOESM4_ESM.tif]

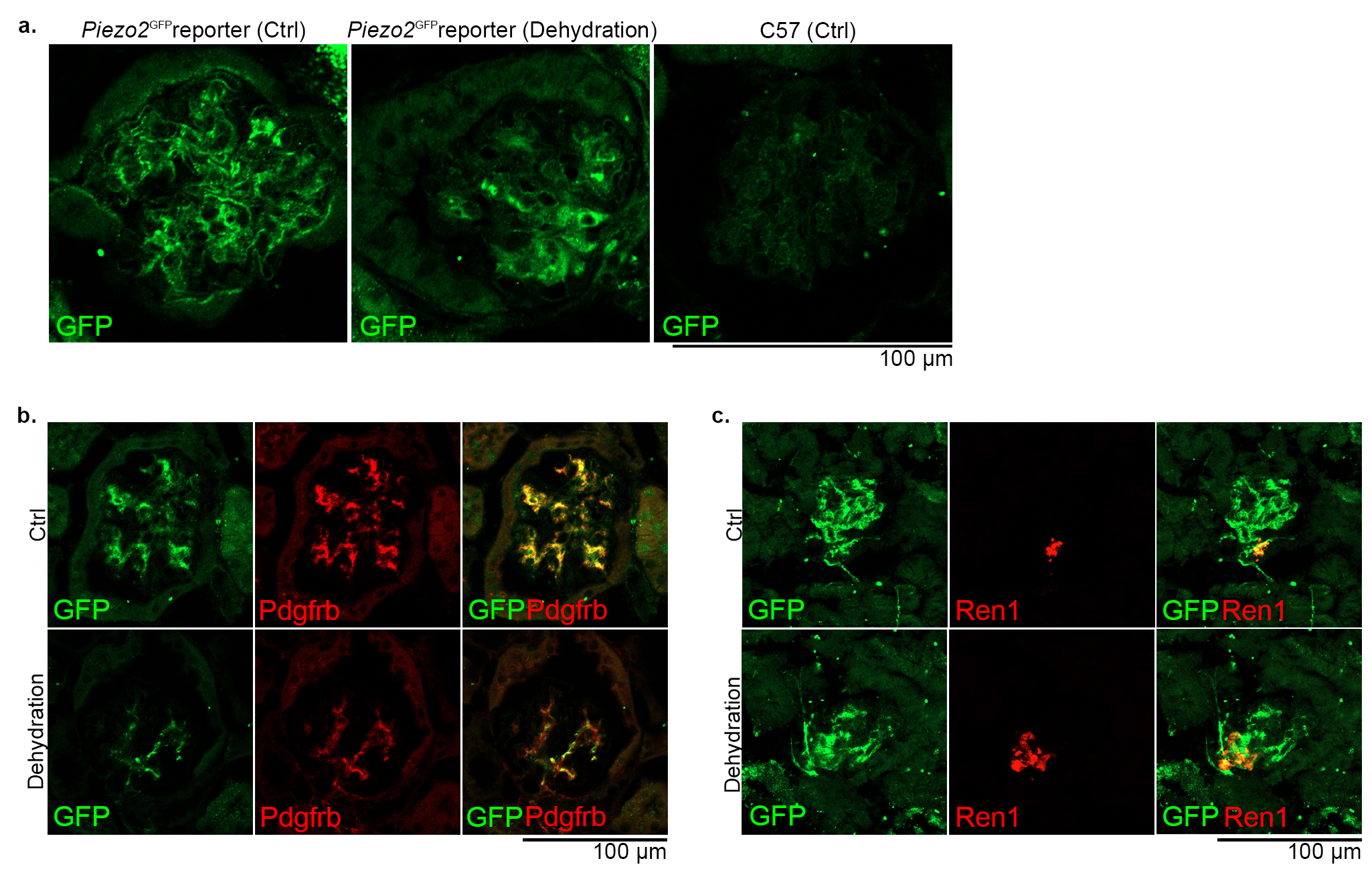

Supplement: Supplementary file 5 — Supplementary Figure S5. [file 41598_2022_7987_MOESM5_ESM.tif]
