# Supplementary material for: Piezo2 expression and its alteration by mechanical forces in mouse mesangial cells and renin-producing cells
Source: Sci Rep. 2022 Mar 10;12:4197. doi: 10.1038/s41598-022-07987-7 (PMC8913706; doi:10.1038/s41598-022-07987-7)
Supplement: Supplementary file 6 — Supplementary Information. [file 41598_2022_7987_MOESM6_ESM.docx]

**Supplementary information**

*Scientific Reports Article*

**Piezo2 expression and its alteration by mechanical forces in mouse mesangial cells and renin-producing cells**

Yuki Mochida^1,2^, Koji Ochiai^1,2^, Takashi Nagase^3^, Keiko Nonomura^4^, Yoshihiro Akimoto^1^, Hiroshi Fukuhara^5^, Tatsuo Sakai^6-8^, George Matsumura^1^, Yoshihiro Yamaguchi^2^ & Miki Nagase^1,7*^

^1^Department of Anatomy, Kyorin University School of Medicine, Tokyo, Japan.

^2^Department of Trauma and Critical Care Medicine, Kyorin University School of Medicine, Tokyo, Japan.

^3^Kunitachi Aoyagien Tachikawa Geriatric Health Services Facility, Tokyo, Japan.

^4^Division of Embryology, National Institute for Basic Biology, Okazaki, Japan.

^5^Department of Urology, Kyorin University School of Medicine, Tokyo, Japan.

^6^ Juntendo University Faculty of Health Science, Tokyo, Japan.

^7^ Department of Anatomy and Life Structure, Juntendo University Graduate School of Medicine, Tokyo, Japan.

^8^ Department of Medical History, Juntendo University Faculty of Medicine, Tokyo, Japan.

*Corresponding author: Professor Miki Nagase, M.D., Ph.D.

Department of Anatomy, Kyorin University School of Medicine, 6-20-2 Shinkawa, Mitaka, Tokyo, 181-8611, Japan.

Tel: +81-422-47-5511, Fax: +81-422-41-5452, e-Mail: mnagase@ks.kyorin-u.ac.jp

**The expanded methods**

**The number of repeats performed and the number of regions observed for RNAscope *in situ* hybridization and immunofluorescence images**

For Fig. 1 (images of RNAscope *in situ* hybridization), we used 6 C57BL/6J mice. RNAscope *in situ* hybridization was performed more than 10 times for characterizing Piezo2-positive cells (searching for cellular markers). As for observed regions, we observed every glomerulus included in the sections. Especially, for *Ren1* staining (**e**), we selected the glomerulus that was cut into maximum cross sections containing renin-producing cells. The most representative images were exhibited as Figures.

For Fig. 2 (images of GFP/cell marker immunostaining), we used 6 Piezo2GFP mice. In total, immunostaining was performed more than 100 times. For GFP, repeated experiments were necessary to determine the optimal condition of antigen retrieval and concentration of primary antibodies. For cell markers, again, we repeated the experiments for selection of primary antibodies (we used 5 Ren1 antibodies) as well as determination of order and condition of antigen retrieval. Every glomerulus was observed in every section. Six C57/BL6 mice were also used because every experiment was performed with negative control. For (**f**), we repeated the “RNA protein Co-Detection Assays” more than 20 times, according to the manufacturer’s instruction, but optimal results were not obtained. Thus, we stained for RNA and protein separately using serial sections.

For Fig. 3 (images of embryos), we created 6 paraffin blocks for each embryonic stage. For RNAscope of embryo samples, experiments were performed more than 10 times to observe what cell markers were colocalized with Piezo2. For quantitative analysis, subcapsular regions were selected as ROI (region of interest) using rectangular tool of ImageJ, and 6 regions derived from 3 embryos were used for each stage. We have indicated *n* = 6 in the Figure Legends.

For Fig. 4 (images of Ctrl and dehydration), (**a**) Kidneys were obtained from 6 Ctrl mice and 6 dehydration mice. Five kidney paraffin blocks from each group were used for RNAscope. (**b**) Four regions (DAB-stained glomeruli were selected as ROI) derived from 4 mice for each group were analyzed for quantitative analysis. (**c**) Six RNA samples from 6 mice for each group were used for quantitative RT-PCR. (**d**, **f**) Double RNAscope for Piezo2/Pdgfrb was performed on 6 samples from 3 mice for each group. Quantification was performed using 6 regions (glomeruli) from 3 mice for each. (**e**. **g**) Double RNAscope *in situ* hybridization for Piezo2/Ren1 was performed on 4 regions (glomeruli containing Ren1-positive area) from 3 mice for each group. Quantification was performed on 4 samples from 3 mice for each group. (**h**) *n* = 2 for each group.

**Figure legends for supplementary figures and videos**

**Supplementary Fig. S1** Validation of RNAscope *in situ* hybridization. (**a, b)** RNAscope *in situ* hybridization and DAB chromogen detection in the mouse kidney. No dot signals are observed for negative control bacterial gene *dapB*, except for a background brown staining (**a**). Dot signals for the positive control mouse housekeeping gene *Ppib* are ubiquitously detected (**b**). (**c-e)** RNAscope *in situ* hybridization and DAB chromogen detection in the mouse nodose ganglion. Hematoxylin and eosin staining (**c**). *Piezo2* expression is clearly detected as brown dots in the ganglion, serving as a positive control (**d**). No signals for *dapB* are observed (**e**). Scale bars: 100 µm.

**Supplementary Fig. S2** Ultrastructural detection of GFP-expressing cells in the *Piezo2^GFP^* mouse kidney by immunoelectron microscopy. GFP immunostaining followed by DAB chromogen detection resulted in brown staining, which was observed as osmium black dots under electron microscope. MC, mesangial cell; RC, renin producing juxtaglomerular cell. (**b**) and (**d**) are enlargement of rectangle areas in (**a**) and (**c**), respectively. Arrows in **b**, **d**:

immuno-positive reaction on the cell membranes. (**e**) and (**f**) are negative control in which the primary anti-GFP antibody was replaced with PBS. RG: renin granules. Scale bars, **a**, **c**, **e** 2 µm; **b**, **d**, **f**, 1 µm.

**Supplementary Fig. S3** Expression of *Piezo2* around the juxtaglomerular *Ren1-*positive region in the Ctrl and dehydration mice. (**a**) Fluorescence double RNAscope *in situ* hybridization of *Piezo2* (red) and *Ren1* (green) in the Ctrl (left) and Dehydration (right) groups. The boxed areas (juxtaglomerular regions) were used to create the 3D images shown in Fig. 4**h**. (**b**) Magnified view of the Ctrl (left) and Dehydration (right) groups from the boxed areas of Fig. S3**a**. Nuclei are stained with DAPI (blue). In addition to *Piezo2-Ren1*-double positive cells, the red dots surrounding the *Ren1* positive area in the Ctrl (left) are colocalized with DAPI, indicating they are not background signals. *Piezo2-*positive cells in the lower-right corner are considered to be glomerular mesangial cells in reference to Fig. S3**a**. Scale bars, **a**, 100 µm; **b**, 20 µm.

**Supplementary Fig. S4** Presumable *Piezo2* expression in extraglomerular mesangial cells. (**a**) Fluorescence double RNAscope *in situ* hybridization of *Piezo2* (red) and *Ren1* (green), together with nuclear DAPI staining (blue), of the same Ctrl section shown in Fig. 4**h** and Fig. S3. Arrows indicate *Piezo2*-positive cells located outside of the arteriolar wall or glomerulus, suggesting that they are extraglomerular mesangial cells. Scale bars, 20µm. (**b**) Fluorescence double RNAscope *in situ* hybridization of *Piezo2* (red) and *Acta2* (a marker for vascular smooth muscle cell, green), together with nuclear DAPI staining (blue) of the C57BL/6J mouse kidney. Longitudinal sections of both afferent and efferent arterioles are seen at the vascular pole. Arrows indicate *Piezo2*-positive cells in the space between the *Acta2*- positive arterioles and renal tubules in the vascular pole, suggesting that they are extraglomerular mesangial cells. Scale bars, upper panels 100 µm; lower panels 20 µm.

**Supplementary Fig. S5** Alteration of Piezo2-GFP protein expression by dehydration in *Piezo2*^GFP^ reporter mice. (**a**) Representative images of Piezo2-GFP protein expression in the kidneys of Ctrl (left) and Dehydration (middle) *Piezo2^GFP^* mice. No signal was observed by anti-GFP immunostaining in the kidney of C57BL/6J mice (right). (**b, c**) Representative double immunofluorescence of GFP (green) and Pdgfrb (red, **b**) and Ren1 (red, **c**) in the kidneys of the Ctrl and Dehydration groups. Scale bars, 100 µm.

**Supplementary Video S1.** Movie of the 3D reconstruction of the double RNAscope *in situ* hybridization images in the juxtaglomerular region of the Ctrl group using Imaris software in the same experiment shown in Figure 4**h**. Z-stack sections were acquired with a Zeiss LSM980 confocal laser scanning microscope for *Piezo2* (red) and *Ren1* (green) by defining the top and bottom focal plane positions and a step size of 1 µm (total 20 focal planes).

**Supplementary Video S2.** Movie of the 3D reconstruction of the double RNAscope *in situ* hybridization images in the juxtaglomerular region of the Dehydration group using Imaris software in the same experiment shown in Figure 4**h**. Z-stack sections were acquired with a Zeiss LSM980 confocal laser scanning microscope for *Piezo2* (red) and *Ren1* (green) by defining the top and bottom focal plane positions and a step size of 1 µm (total 40 focal planes).
